# For Better or Worse: Type I Interferon Responses in Bacterial Infection

**DOI:** 10.3390/pathogens14030229

**Published:** 2025-02-26

**Authors:** Aihong Xia, Xin Li, Changjing Zhao, Xiaojing Meng, Gulmela Kari, Yongjuan Wang

**Affiliations:** 1 College of Veterinary Medicine, Jiangsu Agri-Animal Husbandry Vocational College, Taizhou 225300, China; xah@jsahvc.edu.cn (A.X.); zhaochangjing163@163.com (C.Z.); 2 College of Pharmacy and Chemistry & Chemical Engineering, Taizhou University, Taizhou 225300, China; tc_lixin_0318@163.com; 3 College of Agricultural Economics and Engineering, Kizilsu Vocational Technical College, Kizilsu Kirgiz Autonomous Prefecture 845350, China; mxj_20011026@163.com

**Keywords:** type I interferons, bacterial infection, immune regulation

## Abstract

Type I interferons (IFNs) are pleiotropic cytokines, primarily comprising IFN-α and IFN-β, and their effect in host defense against viral infection has been extensively studied and well-established. However, in bacterial infection, the role of type I IFNs is more complex, exhibiting multifaceted effects that depend on several factors, such as the pathogen species, the specific cell populations, and the routes of infection. In this review, we summarize research progress on host type I interferon responses triggered by specific bacteria and their immune regulation function in order to better understand the role of type I IFNs in bacterial infection and provide insights for adjuvant therapies tailored to treat specific bacterial infections.

## 1. Introduction

**Figure 1:**
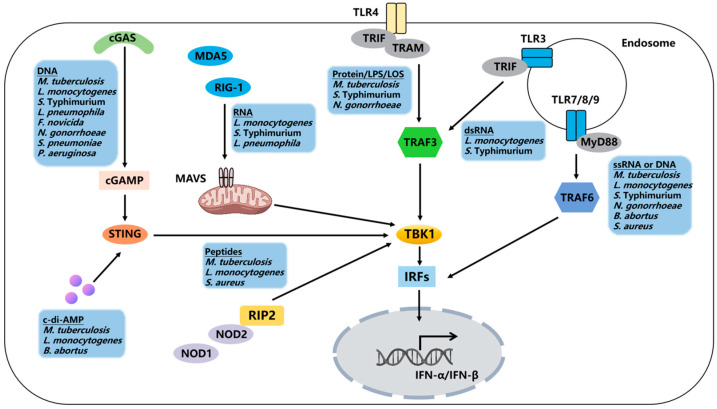
Regulation of the host type I interferon signaling pathway by bacteria. Bacterial DNA/RNA can be recognized by nucleic acid sensors, including cGAS, RIG-I, MDA-5, and TLR3, 7, and 9. Additionally, bacterial components and products can activate type I interferon responses through STING, TLR4, NOD1, and NOD2.

Interferons (IFNs) are a class of proteins or glycoproteins with multiple biological activities, which are divided into type I, type II, and type III [1]. The type I IFN family includes IFN-β, IFN-ω, IFN-κ, IFN-ε, IFN-ζ, IFN-δ, IFN-τ, and 14 different subtypes of IFN-α. The type II IFN family consists only of IFN-γ, while the type III IFN family includes IFN-λ1 (IL-29), IFN-λ2 (IL-28A), IFN-λ3 (IL-28B), and IFN-λ4 [2]. Type I IFNs are generally considered to be immune regulatory factors with antiviral properties as they induce the expression of interferon-stimulated genes (ISGs) [3]. The products of these ISGs can inhibit various stages of viral replication, including blocking viral mRNA synthesis, suppressing viral protein translation, and disrupting virus assembly and release [4]. However, recent studies have shown that type I IFNs also play a critical role in combating bacterial infections, including those caused by *Mycobacterium tuberculosis* (*M. tuberculosis*), *Listeria monocytogenes* (*L. monocytogenes*), *Legionella pneumophila* (*L. pneumophila*), *Salmonella typhimurium* (*S.* Typhimurium), and *Helicobacter pylori* (*H. pylori*). In bacterial infections, the role of type I IFNs is variable, which may exert either beneficial or detrimental regulatory effects [5]. This article summarizes the induction and effects of type I IFNs in specific bacterial infections (Figure 1 and Table 1), and proposes potential intervention strategies to optimize their beneficial effects while minimizing adverse outcomes.

## 2. Type I IFNs Induction and Receptor Signaling

During bacterial infection, the expression of type I IFNs is driven by the interferon regulatory factor (IRF) family of transcription factors, particularly IRF3 and IRF7 [32]. In most cells, IRF3 serves as the primary transcription factor for early type I IFN expression. Subsequently, IRF7 is expressed as one of the ISGs, amplifying the transcription of type I IFNs [33]. Mammalian cells encode numerous pattern recognition receptors (PRRs) that sense invading pathogens and lead to type I interferon production (Figure 1). Upon stimulation of these PRRs, IRFs are activated in a phosphorylation-dependent manner, leading to the induction of type I IFN expression [34]. Bacterial surface components bind to Toll-like receptors (TLRs) 2 and 4 on the cell membrane, while TLR9 anchored in the endosome membrane is activated by bacterial DNA [35]. Single-stranded RNA is sensed by TLR7, TLR8, and TLR13 [36]. Except for TLR3, all TLRs recruit the downstream signal adaptor molecule MyD88 to induce type I IFN expression. TLR4 can recruit both MyD88 and TRIF, but it induces the expression of type I IFNs solely through the TRIF-dependent signaling pathway [37]. RNA is recognized by two RIG-I-like receptors (RLRs), RIG-I and MDA-5 [38]. Peptides from the bacterial cell wall are recognized by NOD1 [31] and NOD2 [39], thereby recruiting the signal adaptor molecule RIP2. DNA is recognized by the cytosolic sensor cGAS, which activates the STING-TBK1-IRF3 pathway through the production of the second messenger cGAMP [40]. In addition to cGAS, DNA can also be recognized by DDX41 and an AIM-like receptor (ALR), IFI16 [41].

Induced type I IFNs bind to their receptor IFNAR1–IFNAR2 heterodimer, activating Janus kinases JAK1 and TYK2. These kinases further phosphorylate STAT family members. Activated STAT1 and STAT2 dimerize and recruit IRF9 to form the ISGF3 complex. The complex subsequently translocates to the nucleus, where it binds to the interferon-stimulated response element (ISRE) in the gene promoter region, thereby initiating the transcription of ISGs [5]. Alternatively, activated STAT monomers form STAT1–STAT1 homodimers, which bind IFN-γ-activated sites (GAS) that promote gene transcription of ISGs [42]. These genes are essential for the activation, proliferation, differentiation, and regulation of inflammatory responses of immune cells [43].

## 3. Type I IFNs in Bacterial Infection

### 3.1. M. tuberculosis

*M. tuberculosis* is the bacterium that causes tuberculosis (TB), which is a leading cause of death from infectious diseases. *M. tuberculosis* can be recognized by host immune cells through surface and intracellular PRRs [44]. Sensing of *M. tuberculosis* infection by TLR4 and TLR9 has been found to induce the expression of type I IFNs [45,46]. Recognition of *M. tuberculosis* is not restricted to TLRs as it can also be detected by NOD2, which activates type I interferon responses in a TBK1- and IRF5-dependent manner in response to bacterial muramyl dipeptide (MDP) [39]. Additionally, *M. tuberculosis* infection triggers mitochondrial DNA release into the cytosol, which subsequently leads to cGAS/STING-dependent induction of type I IFNs [47]. *M. tuberculosis*-secreted cyclic di-AMP (c-di-AMP) can also directly activate STING independently of cGAS [48].

Type I IFNs play a dual role in *M. tuberculosis* infection, promoting both infection and immune defense. The expression levels of type I IFNs induced by *M. tuberculosis* are closely related to the virulence of strains. Compared to *M. tuberculosis* CDC1551, the more virulent clinical isolates HN878 and W4 induce higher levels of IFN-α [49]. This enhanced virulence may be partially attributed to the expression of type I IFNs, which suppresses the production of IL-1β [6]. Interestingly, this effect can be observed in macrophages but not in human monocytes [6]. A mouse infection model indicated that the absence of *IFNAR1* enhances the survival rates of mice infected with *M. tuberculosis* [7]. Similarly, Zhang et al. [50] found that genetic variations in the human *IFNAR1* gene impair type I IFN signaling and reduce susceptibility to *M. tuberculosis*. In addition, some studies reported that sustained and high levels of type I interferon responses exacerbate *M. tuberculosis* infections. Researchers explained the phenomenon by suggesting that type I IFNs can suppress the expression of pro-inflammatory cytokines, such as IL-1α, IL-1β, TNF-α, and IL-12 [8,9,51], while promoting the expression of the immunosuppressive cytokine IL-10 [9], which favors the intracellular survival of *M. tuberculosis*. Type I IFNs can also enhance the spread of *M. tuberculosis* within the host by promoting cell necrosis through the suppression of PGE2 [10]. High levels of type I IFNs not only inhibit the expression of inflammatory cytokines but also impair the host Th1 response, thereby exacerbating the *M. tuberculosis* pathogenic process [52,53]. Although numerous studies support the negative effects of type I IFNs on *M. tuberculosis* infection, there are also reports indicating that type I IFNs may facilitate anti-TB immune responses. A clinical case report suggested that a combination of IFN-α and anti-mycobacterial chemotherapy improves clinical symptoms and reduces bacterial load in patients with active pulmonary tuberculosis who are resistant to conventional treatment or experience disease relapse [11]. Mice lacking both type I IFNs and IFN-γ receptors (*IFNGR1*^−/−^/*IFNAR1*^−/−^) exhibited more severe lung pathology and higher mortality rates compared to mice with a single *IFNGR* deficiency (*IFNGR1*^−/−^) [45]. Further, type I IFNs can enhance the protective efficacy of BCG vaccines by promoting the production of protective cytokines, such as IFN-γ, TNF-α, and IL-12, and increasing resistance to *M. tuberculosis* infection [12]. Generally, researchers agree that sustained high levels of type I IFNs exacerbate TB infection, while early induction of low levels of type I IFNs plays a beneficial role in combating *M. tuberculosis* infection [54,55].

### 3.2. L. monocytogenes

*L. monocytogenes* is a significant foodborne zoonotic pathogen that can penetrate the host intestinal barrier, fetal–placental barrier, and blood–brain barrier, leading to host infection [56]. Clinical symptoms following infection primarily manifest as gastroenteritis, meningitis, sepsis, and miscarriage [57]. After infection of macrophages by *L. monocytogenes*, bacterial RNA is recognized by the cytosolic sensors RIG-I and MDA5 [58], while bacterial DNA activates the DNA sensors IFI16 and cGAS to induce the production of type I IFNs [59]. Cells infected with *L. monocytogenes* secrete extracellular vesicles (EVs) containing bacterial DNA, which fuse with surrounding cells to release the DNA into the cytosol of uninfected cells, thereby activating the cGAS-STING pathway [60]. Type I IFN induction in response to *L. monocytogenes* infection also relies on TLR3 and TLR9 [61,62]. Notably, IFN-β mRNA levels decrease in TLR9-deficient dendritic cells (pDCs) infected with *L. monocytogenes*, while the expression of IFN-α or IFN-β is TLR9-independent in *L. monocytogenes*-infected mice [62]. Furthermore, *L. monocytogenes* MDP can be sensed by NOD2 [63] and *L. monocytogenes*-secreted c-di-AMP can directly activate STING independently of cGAS [64], resulting in the production of type I IFNs.

Early research by T. Fujiki et al. [14] demonstrated that intravenous injection of IFN-β improved the survival rate of mice challenged with *L. monocytogenes* via intravenous injection. Similarly, Elisabeth Kernbauer et al. [13] found that *IFNAR1* is essential for mice to resist *L. monocytogenes* through intragastric infection. However, it is worth noting that bacterial survival increased in *IFNAR1*^−/−^ mice after infection intravenously with *L. monocytogenes* [16], indicating that the route of infection affects the effect of type I IFNs. Recently, more and more studies showed that type I IFNs may exacerbate *L. monocytogenes* infections [65]. The cholesterol-dependent cytolysin, listeriolysin O (LLO), secreted by *L. monocytogenes*, disrupts the integrity of host cellular lysosomes, enabling the bacteria to escape into the cytoplasm [66]. This exposure allows for the recognition of bacterial DNA and RNA by cytosolic sensors. These sensors are subsequently activated, leading to type I IFN-dependent induction of pro-apoptotic genes, such as *DAXX*, *PKR*, and *TRAIL*, which promote apoptosis in macrophages and lymphocytes, thereby facilitating bacterial dissemination and proliferation [16,67]. Consequently, *IRF3*^−/−^ or *IFNAR1*^−/−^ mice exhibit enhanced resistance to *L. monocytogenes* infection, which is attributed to reduced apoptotic cell death, particularly in lymphocytes [16]. During *L. monocytogenes* infection, IFN-α/β also plays a regulatory role in the expression of cytokines. In *L. monocytogenes* infection models, serum levels of IL-12p70 and TNF-α were higher in *IFN-α*/*βR*^−/−^ mice than in wild-type mice [15]. Type I IFNs can exert immunosuppressive effects through an alternative mechanism by downregulating myeloid cell IFN-γ receptor expression. This occurs via the recruitment of an Egr3/Nab1 complex that silences *IFNGR1* transcription. As a consequence, myeloid cells become more susceptible to *L. monocytogenes* infection [17]. In addition, type I IFNs have been found to suppress the host adaptive immune response in *L. monocytogenes*-infected mice. Compared to normal mice, *STING*-deficient mice exhibited restricted bacterial growth and displayed a greater number of cytotoxic lymphocytes upon re-infection [18]

### 3.3. S. Typhimurium

*Salmonellosis* is an important zoonotic infectious disease caused by *Salmonella* spp., primarily characterized by septicemia and enteritis, posing a substantial threat to livestock farming and public health [68]. The host innate immune system senses *Salmonella* infection through different PRRs to initiate type I interferon responses. In non-phagocytic cells, *Salmonella* mRNA is recognized by RIG-1, leading to the induction of type I IFN expression [69]. In phagocytic cells, *Salmonella* dsRNA, LPS, and Curli-DNA are detected by TLR3 [70], TLR4 [69], and TLR9 [71], respectively, resulting in the production of type I IFNs. *Salmonella* can also trigger host cell mitochondrial damage, resulting in the release of mitochondrial DNA, which activates the cGAS-STING signaling pathway and induces the expression of type I IFNs [72].

To investigate the effects of type I IFNs on *S.* Typhimurium infection, peritoneal macrophages were pre-incubated with IFN-β, followed by infection with *S.* Typhimurium SL1344. IFN-β was found to significantly suppress the expression of cytokines IL-1β and IL-18, and the chemokines CXCL1, CXCL2, and CXCL5 [19]. Further, *IFN-β*-deficient mice exhibit enhanced resistance to *S.* Typhimurium infection, with a slower spread of *S.* Typhimurium to sterile sites. Concurrently, higher transcript levels of cytokines and chemokines were observed in *IFN-β*-deficient mice, which facilitate the recruitment and activation of immune cells to more effectively eliminate pathogens [19]. Similarly, Nirmal Robinson et al. found that the survival rate of *S.* Typhimurium was significantly higher in *IFNAR1*^−/−^ mice compared to wild-type mice, and *S.* Typhimurium-induced cell death was inhibited [20]. Induction of cell death by type I IFNs is a crucial pathogenic mechanism employed by *S.* Typhimurium [73]. TLR4/TRIF-dependent IFN-β production is essential for caspase-11 activation, which contributes to macrophage death during *S.* Typhimurium infection [74]. Although cytokine expression and inflammasome activation are not impaired in *IFNAR1*^−/−^ macrophages, they exhibit high resistance to *S.* Typhimurium-induced cell death [20]. Specific inhibition of *RIP1* or knockdown of *RIP3* gene expression can suppress *S.* Typhimurium-induced macrophage death, indicating that *S.* Typhimurium induces cell death via necroptosis [20]. Additionally, the survival ability of *S.* Typhimurium was significantly reduced in *RIP3*^−/−^ macrophages [20]. Thus, *S.* Typhimurium induces macrophage necroptosis by promoting type I IFN expression, allowing the pathogen to evade host immune responses.

### 3.4. L. pneumophila

*L. pneumophila* is a Gram-negative intracellular pathogen that primarily infects amoebae and other protozoa in aquatic environments [75]. However, it can also opportunistically infect humans, particularly those with weakened immune systems, leading to Legionnaires’ disease, a severe form of pneumonia [76]. *L. pneumophila* DNA serves as a primary ligand for inducing host type I interferon responses. Transfection of macrophages with *L. pneumophila* DNA leads to the production of significant amounts of IFN-β [77]. When *L. pneumophila* extract was pre-incubated with DNase I, RNase A, RNase H, and Proteinase K prior to transfection, DNase I could significantly inhibit the secretion of IFN-β induced by *Legionella* extract [77]. During the infection process, *L. pneumophila* DNA is translocated into the host cell cytoplasm through the Dot/Icm-encoded type IV secretion system (T4SS) [78]. Subsequently, *L. pneumophila* DNA is recognized by the intracellular DNA sensor, leading to the production of IFN-β in a STING- and IRF3-dependent manner [21,77]. Meanwhile, *L. pneumophila* RNA can be recognized by RIG-I and MDA5, inducing the expression of type I IFNs [79].

Numerous studies have shown that type I IFNs can protect against *L. pneumophila* infection. Pre-treating mouse bone marrow-derived macrophages (BMDMs) with IFN-α, followed by infection with *L. pneumophila*, demonstrated that IFN-α significantly inhibited the replication of intracellular *L. pneumophila* [21]. Additionally, pre-treatment with IFN-β before *L. pneumophila* infection significantly reduced the intracellular survival of bacteria in BMDMs [77]. Type I IFNs also protect lung epithelial cells from *L. pneumophila* infection in vitro as treatment with IFN-α/β limits the growth of intracellular bacteria [80]. In mouse infection models, *IRF3*^−/−^- and *IFNAR*^−/−^-deficient mice exhibited increased susceptibility to *L. pneumophila* [77], and bacterial loads increased in *cGAS*^−/−^ and *TMEM173*^−/−^ mice intranasally infected with *L. pneumophila* [22] compared to wild-type mice. The molecular mechanisms by which type I IFNs protect the host from *L. pneumophila* infection remain unclear, but they may be related to the promotion of pro-inflammatory cytokine expression [22], the activation of M1 macrophages, and the induction of NO production [21]. Research by Naujoks et al. [81] also suggests that type I IFNs, in conjunction with type II IFNs, alter the composition of bacterial vacuoles by inducing the production of itaconate through IRG1, thereby limiting the replication of *L. pneumophila* in alveolar macrophages and the lungs.

### 3.5. Francisella

*Francisella* is a Gram-negative bacterium, and the most notable species within this genus is *Francisella tularensis*, which causes the disease known as tularemia [82]. This zoonotic pathogen can be transmitted to humans primarily through the bite of an infected arthropod vector, such as ticks, deer flies, or mosquitoes, but also through direct contact with infected animals, inhalation of contaminated dust or aerosols, or ingestion of contaminated water or food [83]. *Francisella* escapes from phagosomes to the cytosol with the assistance of *Francisella* pathogenicity island (FPI) protein IglC and regulator MglA [84]. Once inside the cytosol, *Francisella* dsDNA triggers a type I interferon response through a cGAS- and IFI204-dependent pathway [85], which in turn drives the expression of effector proteins, leading to bacteriolysis and the release of bacterial DNA.

The expression of type I IFNs can promote the expression of absent in melanoma 2 (AIM2) [85], a protein that can bind cytosolic DNA, during *Francisella* infection. Upon recognizing and binding cytosolic dsDNA, AIM2 oligomerizes and recruits ASC [86]. ASC acts as a bridging molecule, further recruiting and activating pro-caspase-1, leading to its self-cleavage into the active form of caspase-1 [87]. Activated caspase-1 can cleave precursor forms of pro-inflammatory cytokines, such as pro-IL-1β and pro-IL-18, processing them into mature, bioactive forms that facilitate their secretion [88]. Additionally, the active AIM2 inflammasome leads to caspase-1-dependent cell death, which helps to restrict pathogen spread and enhance the host immune response [89,90]. At the same time, the IFN-β produced by infected cells serves as a paracrine signal, enhancing the activation of inflammasomes in neighboring cells, thereby facilitating the clearance of *Francisella* before widespread bacterial replication [91]. Paradoxically, type I IFN exacerbates *Francisella* infections. The absence of *cGAS*, *STING*, *IFNAR1*, *IFNAR2,* or *IRF3* has been shown to enhance the resistance of mice to *Francisella* infections [24,85,91]. Qifan Zhu et al. explained that the detrimental effects of type I IFN signaling override the protective responses of the AIM2 inflammasome [24]. In their study, *IFNAR2*^−/−^ mice and *IFNAR2*^−/−^*AIM2*^−/−^ mice were resistant to the *Francisella* infection, whereas all *AIM2^−/−^* mice succumbed to the infection after 12 days. In addition, type I IFNs can suppress host antibacterial responses by inhibiting the expression of IL-17A in γδT cells. In a mouse infection model, *IFNAR1*^−/−^ mice infected with *Francisella* exhibited increased expression of IL-17A, enhanced neutrophil recruitment in the spleen, and improved bacterial clearance and survival rates [23]. Similar to *L. monocytogenes* and *Brucella abortus* (*B. abortus*), type I IFNs also have been shown to participate in TRAIL-mediated apoptosis [24], which increases host susceptibility to *Francisella* infection.

### 3.6. Other Bacterial Infections

The induction of type I IFNs by *Neisseria gonorrhoeae* (*N. gonorrhoeae*) infection is mainly mediated by the TLR4-TRIF-IRF3 and cGAS-STING-TBK-1-IRF3 signaling pathways, with TLR4 recognizing *N. gonorrhoeae* lipooligosaccharides (LOS) and cGAS detecting dsDNA [25,92]. In pDC cells, blockade of the interaction between CpG and TLR9 significantly inhibits *N. gonorrhoeae*-induced type I IFN production, indicating that type I interferon responses depend on TLR9 signaling [93]. Activation of the type I IFN pathway hampers the clearance of *N. gonorrhoeae* as the type I interferon responses can increase iron retention within host cells, creating a microenvironment that favors bacterial survival [25]. Pneumolysin produced by *Streptococcus pneumoniae* (*S. pneumoniae*) causes mitochondrial damage and the subsequent leakage of mitochondrial DNA into the cytoplasm, which activates the cGAS-STING signaling pathway, triggering the production of IFN-β [94]. *IFNAR1*^−/−^ mice exhibited increased bacterial loads following intranasal infection with *S. pneumoniae* [26]. During infection with *Pseudomonas aeruginosa* (*P. aeruginosa*)*,* the cGAS-STING pathway is activated by *P. aeruginosa* DNA to induce type I IFNs [27]. The absence of *cGAS* or *STING* reduced type I IFN production and increased the mortality rate in mice infected with *P. aeruginosa* [27]. *B. abortus* DNA serves as the primary bacterial component that triggers the expression of type I IFNs in a TLR9- and STING-dependent manner, increasing susceptibility to *B. abortus* infection through the suppression of IFN-γ and NO production and the induction of apoptosis [28,95]. However, type I interferon responses were also found to be essential for pro-inflammatory cytokine production, thereby enhancing the resistance of macrophages to *B. abortus* [29]. *Staphylococcus aureus* (*S. aureus*) was sensed by TLR8 and NOD2, leading to the activation of IRF5 and production of IFN-β [96]. Martin et al. [30] demonstrated that a higher survival rate was observed in *IFNAR1*^−/−^-deficient mice infected with *S. aureus* via intranasal administration, which may be partly dependent on the enhanced recruitment of CD11c^+^ DCs. NOD1 can recognize a peptide derived from *H. pylori* peptidoglycan, leading to the induction of type I IFN expression and the subsequent production of CXCL10 [31]. Mice lacking type I IFN receptor or NOD1 are unable to effectively restrict the proliferation of *H. pylori* [97,98].

## 4. Discussion and Future Directions

Type I IFNs induce a powerful antiviral response by upregulating a series of ISGs. In recent years, numerous studies revealed that type I IFNs also play an important role in the host defense against bacterial infection. Unlike their well-defined functions in viral infection, the functions of type I IFNs in bacterial infection are often unpredictable. On one hand, type I IFNs may enhance the host antibacterial immune response by upregulating pro-inflammatory cytokine production, activating the unfolded protein response, reducing bacterial invasion, and inducing M1 macrophage polarization. On the other hand, type I IFNs may also suppress the antibacterial immune response by inducing IL-10 production, inhibiting the expression of pro-inflammatory cytokines and chemokines, and promoting immune cell death (Table 1). The difference in the function of type I IFNs may not only be due to the different preferences of different cell types for the expression of IFN-α or IFN-β and the different affinities for IFNAR, but also due to different infection models, routes, and sites of infection. In the future, we need more in vitro and in vivo experiments to decipher the molecular, cellular, and organismal physiology of type I IFNs in specific bacterial infections, so as to provide new insights for the identification of antibacterial drug targets and vaccine development.

## Figures and Tables

**Table 1:** Effects of type I IFNs during bacterial infection.

Pathogen	Location	Type I IFN Signaling PRRs	Mechanisms/Outcome	Effects of Type I IFNs	Reference
*M. tuberculosis*	Intracellular	cGAS, STING, NOD2, TLR4, TLR9	Limiting the production of IL-1β (macrophages); no effect (monocytes)	Detrimental	[6]
			Promoting intracellular *M. tuberculosis* replication, diminishing alveolar macrophage numbers, and driving tissue damage (aerogenic infection)	Detrimental	[7]
			Reducing TNF, IL-1β, and IL-12 production, and CD54 expression (monocytes); promoting the expression of IL-10 (macrophages)	Detrimental	[8,9]
			Promoting cell necrosis through the suppression of PGE2 (aerosol infection)	Detrimental	[10]
			Combination IFN-alpha-2a with antituberculosis chemotherapy improving a patient’s clinical symptoms	Protective	[11]
			Enhancing BCG-promoted Th1 response (aerosol infection)	Protective	[12]
*L. monocytogenes*	Intracellular	cGAS, STING, NOD2, RIG, TLR3, TLR9	Increasing the mRNA levels of pro-inflammatory cytokines (intragastric gavage infection)	Protective	[13]
			Intravenous injection of IFN-β enhancing host resistance	Protective	[14]
			Inhibiting the production of IL-12p70 and TNF-α (intravenous injection)	Detrimental	[15]
			Enhancing the expression of pro-apoptotic genes and inducing splenic apoptosis (intravenous injection)	Detrimental	[16]
			Downregulating myeloid cell IFN-γ receptor expression (macrophages)	Detrimental	[17]
			Repressing the host adaptive immune response (intravenous injection)	Detrimental	[18]
*S.* Typhimurium	Intracellular	cGAS, RIG, TLR4, TLR3, TLR9	Suppressing the expression of pro-inflammatory cytokines and chemokines, inhibiting the recruitment and activation of immune cells (oral gavage)	Detrimental	[19]
			Inducing macrophage necroptosis (macrophage)	Detrimental	[20]
*L. pneumophila*	Intracellular	cGAS, RIG	Activating M1 macrophages (macrophages)	Protective	[21]
			Promoting pro-inflammatory cytokines expression (intranasal infection)	Protective	[22]
*F. tularensis*/*F. novicida*	Intracellular	cGAS	Inhibiting the expression of IL-17A in γδT cells (intranasal infection)	Detrimental	[23]
			Activating apoptotic caspases and cell death (subcutaneous infection)	Detrimental	[24]
*N. gonorrhoeae*	Extracellular	cGAS, TLR4, TLR9	Enhancing the intracellular iron pool (macrophages)	Detrimental	[25]
*S. pneumoniae*	Extracellular	cGAS	Reducing the invasion of epithelial and endothelial cells (intranasal infection)	Protective	[26]
*P. aeruginosa*	Extracellular	cGAS	Activating the unfolded protein response (intranasal infection)	Protective	[27]
*B. abortus*	Intracellular	TLR9, STING	Suppressing the production of IFN-γ and NO, and inducing apoptosis (intraperitoneal injection)	Detrimental	[28]
			Inducing pro-inflammatory cytokine production (macrophages)	Protective	[29]
*S. aureus*	Extracellular	TLR8, NOD2	Enhancing immune cells recruitment (respiratory infection)	Detrimental	[30]
*H. pylori*	Extracellular	NOD1	Inducing the production of CXCL10 (HT-29 cell)	Protective	[31]
